# The endoplasmic reticulum (ER): a crucial cellular hub in flavivirus infection and potential target site for antiviral interventions

**DOI:** 10.1038/s44298-024-00031-7

**Published:** 2024-06-21

**Authors:** Marijke Verhaegen, Kurt Vermeire

**Affiliations:** KU Leuven, Department of Microbiology, Immunology and Transplantation, Rega Institute, Laboratory of Virology and Chemotherapy, Herestraat 49, 3000 Leuven, Belgium

**Keywords:** Microbiology, Virology, Antivirals, Dengue virus, Virus-host interactions

## Abstract

Dengue virus (DENV) is the most prevalent arthropod-borne flavivirus and imposes a significant healthcare threat worldwide. At present no FDA-approved specific antiviral treatment is available, and the safety of a vaccine against DENV is still on debate. Following its entry into the host cell, DENV takes advantage of the cellular secretory pathway to produce new infectious particles. The key organelle of the host cell in DENV infections is the endoplasmic reticulum (ER) which supports various stages throughout the entire life cycle of flaviviruses. This review delves into the intricate interplay between flaviviruses and the ER during their life cycle with a focus on the molecular mechanisms underlying viral replication, protein processing and virion assembly. Emphasizing the significance of the ER in the flavivirus life cycle, we highlight potential antiviral targets in ER-related steps during DENV replication and summarize the current antiviral drugs that are in (pre)clinical developmental stage. Insights into the exploitation of the ER by DENV offer promising avenues for the development of targeted antiviral strategies, providing a foundation for future research and therapeutic interventions against flaviviruses.

## Introduction

The genus of *Flavivirus* belongs to the family of *Flaviviridae* and includes over 50 arthropod-borne viruses. Several related flaviviruses, including dengue virus (DENV), Zika virus (ZIKV), West Nile virus, Japanese encephalitis virus, tick-borne encephalitis virus and yellow fever virus (YFV) are important human pathogens^1^. DENV is currently considered as one of the top ten global health threats with around half of the world’s population at risk of infection (https://www.who.int/news-room/fact-sheets/detail/dengue-and-severe-dengue). Four genetically related serotypes of DENV (DENV 1-4) co-circulate through transmission to humans by infected *Aedes* mosquitoes, especially *Aedes aegypti*. Currently, DENV is endemic in more than 100 countries in tropical and subtropical regions of Africa, Southeast Asia, the Americas, and the Western Pacific. Global warming, urbanization and intercontinental travelling facilitate the spread of the mosquitos and the virus to non-endemic areas in Europe and the United States^2^.

Infections by DENV are often subclinical, although an estimated 96 million cases each year manifest as dengue illness ranging from a self-limited febrile-like condition (dengue fever) to a life-threatening disease characterized by vascular permeability, severe plasma leakage, shock and organ impairment resulting in 20,000 deaths globally^2–4^. This life-threatening illness is referred to as severe dengue disease^5^. A primary infection with a specific dengue serotype confers lifelong immunity against that particular serotype but only short-term immunity of approximately six months to the other three serotypes. Therefore, severe dengue disease is often associated with a secondary infection with a different DENV serotype through antibody-dependent enhancement which is caused by non-neutralizing antibodies (from the primary infection), making vaccine development extremely difficult as a balanced immune response against all four serotypes is required^2,6,7^. There are currently two approved DENV vaccines commercially available. The first live-attenuated vaccine Dengvaxia, is approved in Brazil, El Salvador, Mexico, Paraguay and the Philippines but with restricted usage due to the lack of efficacy against DENV1 and DENV2 and its related risk of severe dengue disease in seronegative individuals^8^. Recently, another live-attenuated vaccine QDENGA (Takeda) has received its approval in Indonesia, Europe, United Kingdom, and Brazil, however, no efficacy against DENV3 and inconclusive results for DENV4 were reported in seronegative individuals (EMA/862552/2022)^9,10^. Although young age is a risk factor to develop severe dengue disease, QDENGA is only approved for individuals aged 4 and above in the European Union^11^. Major efforts have been invested into vector control strategies such as clearing mosquito breeding habitats, elimination of the *Aedes* larvae, genetic engineering of *Aedes* mosquitos and infecting female mosquitos with Wolbachia some of which are starting to show signs of success^12^. At present, treatment of DENV infection is still restricted to supportive care (antipyretic and fluid supply) as no specific antivirals to treat DENV (and other flavivirus) infection are approved^11^.

This review provides an overview on how flaviviruses, represented mostly by DENV, exploit the endoplasmic reticulum (ER), a key organelle of the host cell, throughout their entire viral life cycle. We briefly describe the flaviviral genome organisation, virion structure and infection cycle before delving into the intricate interplay between the ER and the flavivirus replication cycle at various stages. We cover the molecular mechanisms of viral protein translation, replication, protein processing, and virion assembly in connection to the ER. In addition, we highlight potential antiviral targets in each ER-related step during DENV replication and summarize the current antiviral drugs that are in (pre)clinical developmental stage. Cellular attachment and fusion of flaviviruses are not covered in this review, and potential drugs related to these entry steps are therefore not discussed here.

## Genome organisation and virion structure

Flaviviruses share a common genomic organisation consisting of a 11 kb positive-single-stranded RNA molecule containing a single open reading frame (ORF) flanked by a 5′ and 3′ untranslated region (UTR). The ORF encodes for a single polyprotein that is processed into three structural proteins [capsid (Ca), membrane (M) and envelope (E) protein] and seven non-structural (NS) proteins (NS1, NS2A, NS2B, NS3, NS4A, NS4B and NS5). The Ca protein units assemble into a spherical core and interact with the viral genome to form the nucleocapsid^13^. The nucleocapsid is surrounded by a host-derived membrane in which the E and M glycoproteins are embedded. The conformation of both structural glycoproteins differs in immature and mature viral particles^14,15^. The E protein is the major protein on the surface of the flavivirus virions and mediates viral entry and fusion. More specifically, the protein is composed of three main domains: EDI, the structurally central domain containing the N-terminus; EDII, the internal fusion domain; and EDIII, the receptor binding domain at the C-terminal side and the major target for neutralizing antibodies. The premature form of the M protein, referred to as the pre-M (prM) protein, is associated with the E protein on the viral surface and prevents the latter from undergoing premature activation into a fusion competent state. The prM protein also assists in the chaperon-mediated folding of the E protein during its biogenesis^13,16^.

The NS proteins are not incorporated into the mature virions but are expressed in the infected cells where they are involved in viral replication, assembly and evasion of the host cell’s immune response. The NS1 protein exists in different forms, including a dimeric form within the host cell, a membrane-anchored form on the cell surface, and a secreted (hexameric) form. Intracellular NS1 is involved in replication and virus maturation. The secreted NS1 is the most pathogenic DENV protein as it has been linked to endothelial dysfunction and immune evasion^17,18^. NS2A is a small hydrophobic membrane-associated protein with distinct roles in viral replication and assembly^19^. NS2B, also a small membrane-embedded protein, forms a complex with NS3 and acts as a cofactor in the NS2B-NS3 serine protease complex^20^. NS4A and NS4B are transmembrane proteins containing three and five transmembrane domains (TMDs), respectively. However, TMD2 of NS4A and the two N-terminal TMDs (1 and 2) of NS4B are weakly hydrophobic helices which are positioned in the luminal leaflet of the endoplasmic reticulum (ER) membrane rather than spanning the membrane^21–23^. Both proteins support viral replication by orchestration of the replication platform at the ER^24^. The 2 K peptide between NS4A and NS4B is not present in any of the mature NS proteins but functions as a cleavable localisation signal for NS4B (signal peptide-like targeting sequence for the ER)^22^. NS3 and NS5 are the largest flavivirus proteins and possess multiple enzymatic activities. NS3 contains an N-terminal protease domain and a C-terminal triphosphatase and helicase domain involved in viral genome capping and unwinding of the viral RNA, respectively. The N-terminal domain of NS5 is a methyltransferase involved in 5′ RNA capping and cap methylation, whereas the C-terminal portion of NS5 is an RNA-dependent RNA polymerase (RdRp). NS3 and NS5 are both soluble (cytosolic located) proteins without TMD and can be retained at the site of replication (at the ER membrane) through binding to the viral transmembrane proteins^1,13,25^.

## Infection cycle

**Fig. 1:**
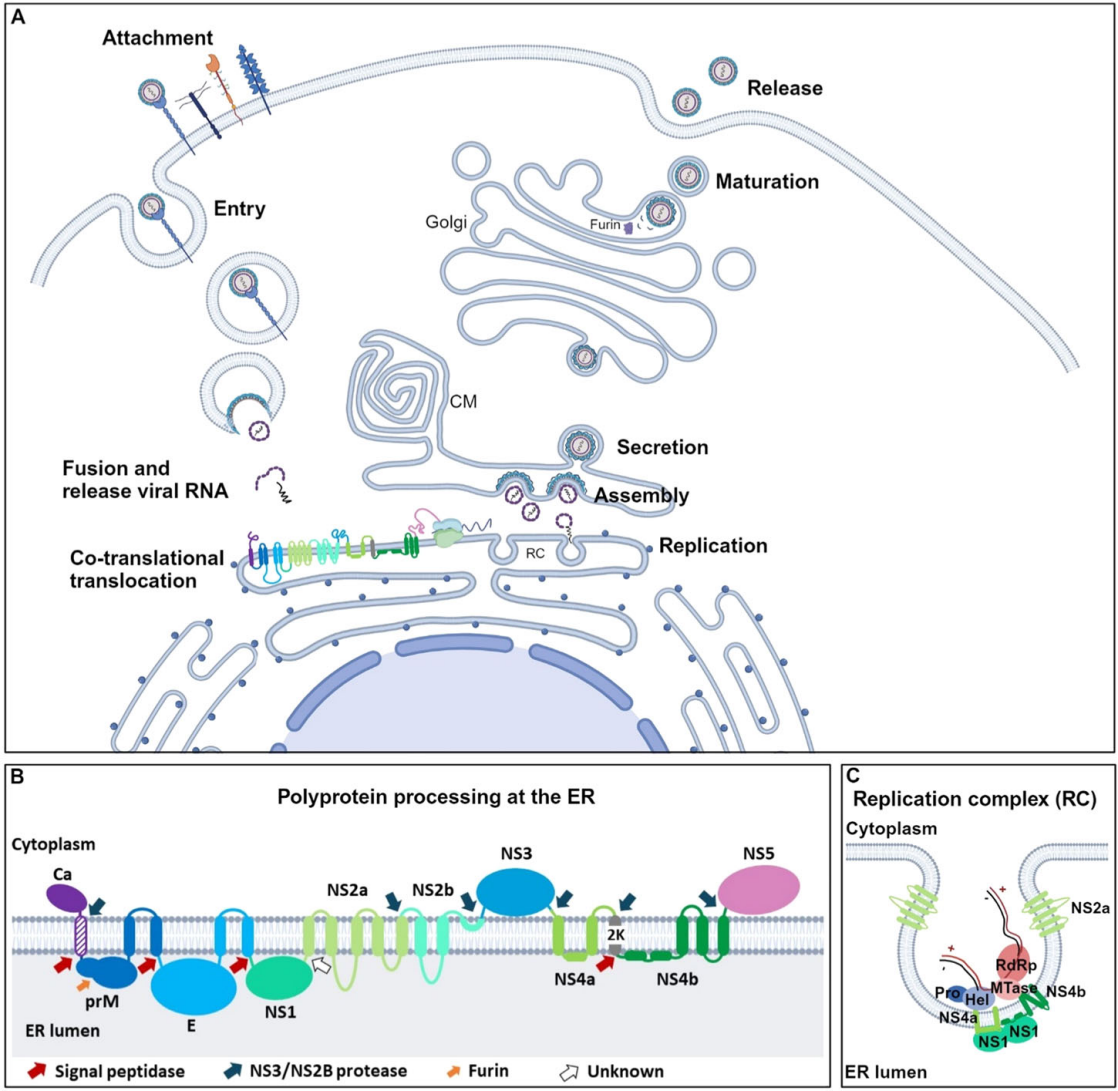
The role of the endoplasmic reticulum (ER) in the dengue virus (DENV) life cycle. **A** DENV (flavivirus) life cycle. DENV can attach to a broad range of receptors and enter the cell via clathrin-mediated endocytosis. Subsequent fusion of viral and endosomal membranes results in release of the viral RNA genome. The RNA is translated into one large polyprotein that is inserted at the ER membrane where it is further processed into its individual structural (Ca: capsid, E: envelope and prM: pre-membrane) and non-structural (NS1, NS2A, NS2B, NS3, NS4A, NS4B, NS5) proteins. The NS proteins orchestrates the assembly of the replication complexes (RC) and replicate the viral RNA genome. The ER is a large and highly interconnected membranous network that extends from the nuclear envelope. The ER cisternae that harbour RCs would be interconnected with the ER that harbours the virion assembly sites. The newly generated viral RNA complexes with the Ca protein units and the resulting nucleocapsid is engulfed by the viral membrane (containing prM and E proteins) through budding into the ER lumen, predominantly in regions opposite to the RC. Maturation of new virions by furin-mediated cleavage of prM proteins occurs along the secretory pathway within the Golgi apparatus. CM: Convoluted membranes. **B** DENV polyprotein topology and predicted transmembrane domains at the host cell’s ER membrane. The polyprotein is processed by cellular signal peptidase at the ER lumen and by viral protease (NS2B/NS3) at the cytoplasmic side. The prM proteins further mature by furin cleavage and the additional NS1/NS2A cleavage is carried out by a yet unknown enzyme. **C** Model of the DENV-induced RC at the ER membrane. NS4A and NS4B might induce a negative membrane curvature which is stabilized by the homo- and hetero-oligomerization of NS4A and NS4B (not depicted) along with the association of NS1 at the luminal side of the ER. NS2A and other host factors (not depicted) may also contribute to the membrane reformations. RdRp: RNA-dependent RNA polymerase, MTase: NS5 methyltransferase, Hel: NS3 Helicase, Pro: NS2B-NS3 viral protease, +: plus RNA strand (red) and −: minus RNA strand (black). The figure was created with Biorender.com.

Similar to several other enveloped viruses, the infection cycle of flaviviruses (see Fig. 1A) starts with the attachment of the viral particles to a structurally diverse group of receptors on the host cell. For DENV e.g., these include the dendritic cell-specific intracellular adhesionmolecule-3-grabbing non-integrin (DC-SIGN), glycosaminoglycans such as heparan sulfate, mannose receptors, heat-shock proteins (HSP) 70 and 90 and immunomodulatory proteins (TIM/TAM receptors)^26^. The virus-receptor complex enters the host cell via clathrin-mediated endocytosis. After internalisation, the low pH of the endosome initiates dissociation of the E dimers on the viral envelope into monomeric E intermediate with the fusion loop exposed (fusogenic form) that allows for fusion of viral and endosomal membranes^12^. Next, the viral RNA is released into the cytoplasm where it serves as mRNA for translating ribosomes. These ribosomes are targeted to the ER membrane of the host cell to secure the translation of the viral polyprotein that gets integrated into the ER membrane. The viral polyprotein is co-and post translationally processed (cleaved) into its individual structural and non-structural proteins which adapt their mature state by further folding and glycosylation. Next, the NS proteins orchestrate the formation of the replication complexes (RC) at the ER membrane where large amounts of progeny positive strand RNA molecules are synthesized by the viral polymerase. During encapsidation, each viral RNA molecule is packaged into a de novo synthesized particle that eventually buds from the ER. The virions are transported within vesicles along the secretory pathway where they undergo the final maturation before being released from the infected host cell by exocytosis^1,24^.

## Flaviviruses and the ER

The key organelle in flaviviral (and in many other viral) infections is the host cell’s ER which supports different steps throughout the whole flaviviral life cycle. The ER is a complex organelle that plays a pivotal role in protein and lipid synthesis, calcium storage and stress response. It provides numerous channels, chaperones, enzymes, as well as a favourable membrane platform for biogenesis of the flavivirus RCs owing to its expansive surface area and its ability to encounter continuous membrane rearrangements^27^. In the context of flavivirus infections, the ER also provides different opportunities/target sites for anti-flaviviral drug development.

### The ER

The ER is the largest and a vital organelle in the eukaryotic cell and is composed of a continuous array of membranous network undergoing constant rearrangements. One of the main functions of the ER is to support biosynthesis of extracellular/secreted and transmembrane proteins, comprising one-third of the entire cellular proteome^27^. The main protein conducting channel in the ER membrane is the Sec61 translocon, a 10-helical protein complex composed of three subunits: SEC61α, SEC61β, and SEC61γ. During co-translational translocation, a process in which translation of the protein is coupled to its transport across or insertion into the ER membrane, the translating ribosome in the cytosol is guided towards the Sec61 translocon in the ER membrane with the support of the signal recognition particle (SRP) and its receptor (SRR) on the ER membrane. N-terminal signal peptides or internal hydrophobic TMDs are the targeting sequences of the preprotein for co-translational ER translocation. These are recognized by SRP that docks the ribosome/nascent chain complex onto Sec61^28–30^. In contrast to the TMDs, N-terminal signal sequences are not part of the mature protein but are cleaved from the preprotein (while being translocated into the ER) by a signal peptidase (SPase) that resides in the ER lumen^31,32^. Secretory proteins are translocated across the ER membrane and released into the ER lumen (vertical transport along the Sec61 translocon). On the other hand, membrane-anchored proteins with one or more hydrophobic TMDs undergo integration into the ER lipid bilayer during the translation process through lateral movement of the TMD along the ‘lateral gate’ of the Sec61 channel (lateral insertion in ER membrane)^28–30,33^. The translocon-associated protein (TRAP) cooperatively directs the synthesis of the nascent polypeptides ensuring their correct topology in the ER membrane^34^. Multi-pass proteins with multiple TMDs and complex topologies often rely on other chaperone mechanisms (such as the ER membrane protein complex [EMC] or the PAT complex) for insertion of their diverse TMDs^35,36^. Finally, the translocated proteins are further post-translationally modified in the ER, such as N-glycosylated by the oligosaccharyltransferase (OST) complex to assist their proper folding and function^37^. For an overview of co-translational translocation, we refer to the review by Pauwels and colleagues^38^.

In addition to protein synthesis and quality control, the smooth ER is the site of lipid synthesis (cholesterol, fatty acids, and phospholipids), important components of cell membranes and cellular signalling. Finally, the ER is intricately connected to other cellular compartments by the endomembrane network from which (COPII protein coated) vesicles bud that carry the correctly folded and assembled proteins or lipids to various cellular compartments (Golgi or lysosomes) or to the plasma membrane (transmembrane proteins)^27,39^.

### Translation of the flavivirus RNA genome

After release of the flavivirus genome into a newly infected host cell, the translation initiation is likely to start in the cytosol before the ribosome is targeted towards the ER. The 5′ and 3′ UTR of the flaviviral genome contain multiple sequences and secondary structures essential to regulate translation and replication of the viral genome^1^. The flavivirus RNA contains a 5′ type I 7-methylguanosine cap and therefore initiation can be accomplished in a cap-dependent way. The eukaryotic translation initiation factor (eIF) 4E, the mediator of cap-dependent translation, binds to the cap and orchestrates the formation of the 80S elongation competent ribosome complex. Alternatively, DENV can hijack a non-canonical translation initiation when cellular conditions do not support any cap-dependent initiations. Translation initiation is likely supported by structures in the 3′ UTR and their interaction with host factors such as eIF1A, poly-pyrimidine tract binding protein, y-box binding protein 1 and nuclear ribonucleoproteins A1, A2/B1 and Q and Poly(A) binding protein (despite the lack of a 3′ poly A tail)^40^. Elongation of the viral polyprotein is supposed to occur via a non-canonical mode as shown by its strong dependency on the ribosome stalk-associated acidic phosphoproteins RPLP1 and RPLP2^1^.

Several antivirals that directly inhibit viral translation have been reported. Peptide-conjugated phosphorodiamidate morpholino oligomers (PPMO, nucleobases attached to a backbone of morpholine rings), complementary to the 5′ and 3′ UTR of the viral genome, can inhibit DENV translation by sterically blocking RNA-RNA and RNA-protein interactions required for the formation of the 43S preinitiation complex^41^. As the sequences of the viral UTR’s are divergent, PPMO’s are relatively virus-specific and well-tolerated but have limited potential as broad-spectrum antivirals^42^. If administered before virus exposure, PPMO’s treatment in DENV-challenged AG129 mice was effective while no activity was measured in mice receiving a post-infection treatment^43^. Inhibition of DENV translation was also proposed as the mode of action for the benzomorphan NITD-451. However, this compound showed non-selective inhibition of viral and host RNA translation. NITD-451 treatment reduced viremia in DENV-infected mice but also resulted in adverse effects at a higher dose (75 mg/kg)^44^. The natural product lactimidomycin, an inhibitor of translation elongation via ribosomal binding, was reported to exert an anti-flavivirus activity^45^. Other inhibitors of viral protein translation are the synthetic flavonoid ST081006 and the ionophore narasin^46,47^. The enzyme inhibitor AR-12 which might inhibit phosphatidylinositol 3 kinase (PI3K)/AKT-mediated viral translation also showed promising anti-DENV potency. PI3K/AKT promotes the formation of eIF4E complex via mTOR, a pathway shown to be activated in DENV infection to promote replication^48^.

### Flavivirus protein translocation at the ER

Irrespective of whether flavivirus translation initiates in the cytosol by free ribosomes or on ER-associated ribosomes, polyprotein synthesis takes place in association with ER membranes. Genetic screens identified components of co-translational translocation, such as SRP9/54/14, TRAP subunits SSR1/2/3, and Sec61/63, as essential flavivirus host factors in human and vector, highlighting the importance of this cellular process for flaviviral replication^49–53^. The newly translated flavivirus polyprotein is likely targeted towards and incorporated into the host cell’s ER membrane by co-translational translocation in a similar way as for cellular transmembrane proteins. The DENV polyprotein contains 18 TMDs, four of them resembling a signal peptide-like targeting sequence containing a cellular SPase proteolytic cleavage site (see Fig. 1B). These four targeting sequences, namely the TMD at the COOH-terminus of the Ca protein (which is the first TMD that emerges from the ribosome exit tunnel and gets recognized by SRP), the C-terminal TMD of prM, the C-terminal TMD of the E protein, and the 2 K peptide, direct translocation into the ER of each downstream polyprotein subunit (i.e., M, E, NS1 and NS4B, respectively)^22,54–56^. Comparable to signal peptides of cellular proteins, the TMD at the COOH-terminus of the Ca protein serves as an ER localization element for the ribosome/nascent chain complex. Its interaction with SRP arrests translation and targets the ribosome generating the nascent polypeptide to the Sec61 translocon in the ER membrane. Once the ribosome has docked onto the Sec61 translocon channel, translation continues, and the polyprotein is embedded into the ER membrane as a multi-pass transmembrane protein, with the pr-M, E, NS1 and some extended stretches of NS2A, NS2B, NS4A and NS4B located at the lumenal side of the ER membrane whereas the Ca, NS3 and NS5 proteins are positioned at the cytoplasmic side of the ER. The more hydrophobic domains of the Ca, prM, E, NS2A, NS2B, NS4A and NS4B proteins undergo lateral insertion into the ER membrane to become the TMDs of the polyprotein and the final membrane anchors of the different cleaved subunits (Fig. 1B)^1^.

Interestingly, the EMC was identified as another flavivirus dependency factor^49,57,58^. The EMC consists of a six-subunit core complex (EMC 1-6) which has been shown to act as a multi-pass transmembrane chaperone. It has been suggested that it functions as membrane insertase for tail-anchored and multi-pass proteins (e.g., G protein-coupled receptors)^59–62^. The EMC specifically stabilizes multi-pass membrane proteins that have difficulty to engage with the Sec61 translocon based on their intrinsic features (such as length, hydrophobicity and flanking charged regions), thereby helping to avoid misfolding and degradation of nascent protein chains^60^. In case of DENV, two independent studies discovered that the biogenesis of NS4A and NS4B proteins, two multi-pass TM proteins, depends on the EMC. The EMC was shown to interact with NS4B during ER translocation, to act as a NS4B chaperone by facilitating its folding and correct topology at the ER membrane but not its post-translational stability^63,64^. The N-terminal intramembrane domains of the NS4B protein were found to confer the EMC dependency^63^. Their weak hydrophobicity likely impedes the proper integration into the ER membrane during biosynthesis and therefore NS4B may require the chaperone function of EMC for successful membrane insertion and to avoid protein degradation^63,64^. EMC does not affect the ER targeting and translocation of the 2 K peptide region indicating that more hydrophobic TMDs of the DENV polyprotein (such as 2 K) do not rely on EMC chaperone assistance to the Sec61 translocon. However, EMC dependency of NS4B is linked to the presence of NS4A as protein destabilisation in EMC KO cells was only observed when NS4B was preceded by NS4A^64^. Together, this demonstrates that the biogenesis and the co-translational stability of flavivirus multi-pass proteins, including NS4B, relies on EMC but the exact interplay between the Sec61 translocon and EMC for the flavivirus polyprotein remains to be elucidated. Intriguingly, the EMC subunit 4 was also shown to promote fusion of the DENV and endosomal membranes to enable the release of the viral RNA into the cytosol. More specifically, EMC4 was shown to promote the transfer of phosphatidylserine, a phospholipid which facilitates DENV-endosomal membrane fusion, from the ER to the endosomes through stabilization of their contact site^65^. This indicates that an ER-localized host factor can also be involved in endosomal membrane fusion thereby regulating an early entry step of DENV infection.

Several inhibitors of Sec61-dependent protein translocation have been described, which can be considered as promising anti-cancer, immunosuppressive, and antiviral therapeutics^38^. Although the Sec61-dependent translocation complex was identified as an important host factor for flaviviruses in multiple screens^49–52^, inhibition of this ER-related step has not garnered much attention regarding its potential for pan-flavivirus drug development thus far. In two recent studies, Heaton et al. and Shah et al. demonstrated inhibition of DENV replication by the Sec61 translocon inhibitor cotransin 8, and its analogue PS3061, in monocyte-derived dendritic cells and vector cells^53,66^. Apratoxin S4 is another Sec61 inhibitor for which a highly potent pan-flaviviral activity (DENV2, ZIKV and WNV) was reported^67^.

### Proteolytic cleavage of the flavivirus protein by signal peptidase

Translation and correct insertion into the ER membrane is followed by coordinated processing and cleavage of the precursor polyprotein into the separate flaviviral proteins by host cell SPase in the ER lumen and by the viral protease (NS3 in complex with cofactor NS2B) at the cytosolic side of the ER membrane. Host cell SPase is a membrane-bound serine protease complex composed of three accessory proteins (SPCS1-3), and two proteolytic subunits (SEC11A and SEC11C) which cleaves the signal peptides of newly synthesized secretory and membrane proteins^32^. Accessory proteins SPCS1 and SPCS3 were identified as essential flavivirus host factors in genetic screens. SPCS1 knock-out cells did not support Ca-prM, prM-E, E-NS1 and 2K-NS4B cleavages indicating their SPCS1 dependency^51^. The N-terminus of prM, E, NS1, and NS4B is thus released from its upstream partner upon cleavage by the host SPase in the ER lumen^51,55^. Given its significance in the flavivirus life cycle, SPase presents an attractive target for the development of an antiviral drug. An example of an SPase-inhibitor is the natural compound cavinafungin which inhibits infection of the four DENV serotypes and ZIKV. Estoppey and colleagues hypothesize that the oligopeptide moiety of cavinafungin behaves as a signal peptide and interacts with the peptide binding cleft of the SEC11A subunit, resulting in a selective inhibitory effect on the eukaryotic SPase. Conferring mutations in the SEC11A signal peptide binding cleft and the critical importance of the aldehyde group of cavinafungin for its activity supported this hypothesis^68^. The down-side of such an inhibitor might be the considerable cellular toxicity related to the inactivation of a central protease involved in the processing of many cellular substrates.

### Proteolytic cleavage of the flavivirus protein by viral protease

The N-terminal side of NS3 forms the catalytic domain of the NS2B-NS3 trypsin-like serine protease complex, i.e., the viral protease which is responsible for cleavage at the NS2A/NS2B, the NS2B/NS3, the NS3/NS4A and the NS4B/NS5 junctions (Fig. 1B). Moreover, the protease is responsible for release of the mature Ca protein and NS4A protein at the C-terminus and has the capacity to cleave at internal sites within NS2A and NS3. The remaining proteolytic cleavage of the viral polyprotein between NS1 and NS2A at the lumenal side of the ER is performed by a still unknown protease. The substrate binding residues of the viral NS2B-NS3 protease are well conserved among flaviviruses and provide a promising target for the development of broad-spectrum antivirals. The DENV NS2B-NS3 protease has preference for substrates with dibasic or polybasic residues (e.g., arginine and lysine) in the recognition sequences^42^. In silico molecular docking and high-throughput screenings have been popular approaches for the discovery of potential NS2B-NS3 protease inhibitors. Numerous peptidomimetics mimicking NS2B-NS3 protease substrates were reported with potent in vitro anti-DENV protease activity, but only few of them showed potency in DENV-infected cell cultures (see Table 2). Alternatively, non-peptide small molecule inhibitors of natural (e.g., protegrin-1, palmatine and flavonoids) or synthetic origin with inhibitory effect on the DENV NS2B-NS3 have been reported (Table 2)^69^. Several small molecules with inhibitory activity in the (sub)-micromolar range against the DENV protease and DENV replication in cell culture were identified (examples are diaryl(thiol)ethers, SK-12 and BP2109; see Table 2). Nevertheless, two decades of intensive research has revealed that the flavivirus protease is not an easy target. One of the main challenges is that the active site of the viral protease greatly resembles cellular serine proteases resulting in adverse effects when blocking NS2B-NS3^69^. In contrast, targeting the viral protease of the related HCV or of HIV has been successful. Furthermore, HCV and HIV protease inhibitors have been exploited as a source for repurposing drugs against flaviviruses. Interestingly, the anti-HIV drug nelfinavir showed potency against DENV in vitro with low cellular toxicity^70^. Furthermore, several recent anti-cancer molecules turned out to be potent inhibitors of the DENV NS2B/NS3 protease as well. More specifically, four B-cell lymphoma-2 inhibitors from a library of structurally diverse small anti-cancer molecules exhibited the highest potency against the DENV NS2B/NS3 protease^71^. The repurposed anti-cancer drugs sunitinib and erlotinib are discussed in the section on assembly and virion budding.

### N-linked glycosylation of the flavivirus proteins

Protein glycosylation, catalysed by the ER-resident OST complex, is another essential step in the replication cycle of flaviviruses given that glycans contribute to the native structure and stability of the viral envelope and some NS proteins, and to viral escape from the humoral immune response. Asparagine (N)-linked glycosylation sites have been identified on the prM, E, NS1 and NS4B protein^13,72,73^. Multiple studies demonstrated that DENV replication depends on both mammalian OST isoforms, which are multiprotein complexes with paralogue catalytic subunits (STT3A and STT3B)^49,72,74^. However, these studies have different views on the enzymatic function (N-linked glycosylation) of the OST complex. Hafirassou et al. claimed in favour of the N-linked glycosylation function of OST as a requirement for DENV replication^72^. On the contrary, studies by Marceau et al. and Lin et al. dismissed a role of the OST-dependent N-glycosylation in viral replication but suggested that DENV replication depends on a structural role of the OST complex (see later section) or on the oxidoreductase activity of magnesium transporter protein 1, the non-canonical OST complex subunit, respectively^49,74^. The small molecule inhibitor NGI-1 that targets the OST complex was reported to inhibit DENV1-4 infection at (sub-)micromolar concentrations^72,75,76^. While Hafirassou et al. contributed this inhibition to reduction in NS1 N-linked glycosylation, Puschnik et al. suggested that NGI-1 blocks viral replication independent of the OST catalytic activity but rather through inhibition of direct OST usage by the viral replication machinery^72,76^. The latter would explain the low cellular toxicity associated with NGI-1 as it will not compromise cellular glycosylation. Another promising factor regarding this drug is its low risk to the development of viral resistance because four amino acid changes in the viral genome were identified that could explain escape to NGI-1 treatment^76^.

### Trimming of N-linked glycosylation

Regardless of the role of OST in the biogenesis of the flavivirus proteins, initial glycosylation is followed by sequential trimming of the oligosaccharide precursor by ER α-glucosidase I and II enzymes. Trimming of three glucose residues enables the glycoproteins to engage with ER chaperones like calnexin and/or calreticulin, ensuring their correct folding^73^. Glucosidase inhibitors castanospermine and deoxynojirimycin, both iminosugars, exert broad-spectrum anti-(flavi)viral activity and their administration results in misfolding of the glycoproteins prM, E and NS1^77,78^. Celgosivir, a more potent prodrug of castanospermine, is active against all four DENV serotypes and was shown to limit the activation of the host’s unfolded protein response (UPR) caused by DENV infection^79^. In spite of its low toxicity and protective effect in AG129 mice, celgosivir failed in proof-of-concept clinical trials with patients exhibiting dengue fever symptoms (NCT01619969), however, it is currently under investigation in a new clinical trial with a revised dosing regimen (NCT02569827)^80,81^. Another α-glucosidase inhibitor, N-(9′-methoxynonyl)-1-deoxynojirimycin, or UV-4B, proofed to be safe and well-tolerated in healthy subjects (NCT02696291) but was not further explored in follow-up studies^82^. Many other deoxynojirimycin analogues with anti-flaviviral activity in vitro, especially against DENV, have been reported within the last two decades. Several deoxynojirimycin analogues also protected against DENV infection in vivo^83–89^. Furthermore, the less studied valiolamine derivates inhibit α-glucosidase I and II up to 1500-fold higher level than the benchmark UV-4 by occupying all four substitutes in the active site of the enzyme and were at least as effective as UV-4 against DENV in vitro^90^. Klein and colleagues considered the use of glucosidase inhibitors as emergency prophylaxis because they can act as broad-spectrum antivirals by targeting a host factor early after viral infection^42^.

### Flavivirus protein folding

The complex topology of the flavivirus polyprotein implies the support by multiple ER-resident chaperones and the need of chaperone like-activities during its biogenesis^91^. The role of the ER-resident EMC chaperone in the biogenesis of NS4A and NS4B was already discussed above. Also, heat shock protein 70 (HSP70) family of chaperones and a subset of its co-chaperone DNAJ homologue subfamily support viral replication or particle biogenesis. HSP70 was found to be important for folding and stabilization of the Ca and NS5 protein and localizes in the RCs where it contributes to NS5 activity. The HSP70-DNAJ network provides an opportunity for host directed antivirals. JG40, an inhibitor of the HSP70-DNAJ network exerted an anti-DENV (2-4) activity with high barrier to resistance development and low cellular toxicity^92^. In addition, host chaperones such as calreticulin, calnexin and GRP78 (BiP) have been shown to facilitate correct folding of the viral surface E glycoprotein after their synthesis at the ER^93^. The earlier mentioned celecoxib derivative AR-12 was shown to downregulate chaperone GRP78 and is active against the four DENV serotypes and reduces DENV-induced mortality in mice^48^.

### Unfolded protein response in flavivirus infection

Accumulation of misfolded proteins and related ER stress evokes the UPR. UPR is an intracellular signalling cascade to counteract with ER stress and avert cellular autophagy and is accompanied by increased ER protein folding, reduced mRNA production and transcriptional activation of genes encoding chaperones, oxidoreductases, and ER-associated degradation (ERAD) components. ERAD is a highly conserved quality control mechanism that targets and transports misfolded proteins to cytoplasm for ubiquitin-dependent degradation by the proteasome. Soluble misfolded ER proteins are exported to the cytosol through the Hrd1-Derlin1 ER membrane channel, while large misfolded proteins are removed by ER-phagy, an autophagic pathway that selectively degrades portions of the ER^27,39^. It must be noted that the presence of viral proteins triggers an intracellular stress response in infected cells. UPR-related pathways inositol-requiring transmembrane kinase/endoribonuclease 1 (IRE1), protein kinase R (PKR)-like endoplasmic reticulum kinase (PERK) and activating transcription factor-6 (ATF6) are modulated in DENV infection as reviewed by Das and colleagues^39^. Expectedly, genetic screens identified components of the ERAD pathway (Derlin 2, SEL1L, UBE2J1, NSFL1 C, UBE3A, UFDL1 and AUP1) as important for flavivirus replication^49,52^. The molecular mechanism behind this involvement remains largely unknown. NS2A, NS4A and NS4B were shown to be degraded by the valosin-containing protein (VCP)/Derlin2-mediated ERAD pathway. Probably, the synthesis of a single polyprotein results in the production of equimolar amounts of the individual proteins, thus, in an excess of viral NS proteins relative to structural proteins, which might disturb viral homeostasis. The excess of NS proteins is discarded by ERAD^94^. Furthermore, it has been shown that two ERAD-related E3 ligases, i.e., HRD1 and RNF126, mediate ubiquitination of NS3. Ubiquitination of ZIKV NS3 negatively affected its viral protease activity. It has also been suggested that HRD1 ubiquitinated both misfolded DENV and ZIKV NS3 proteins which are retro-translocated into the cytosol for proteasomal degradation, and meanwhile trimmed, thus, requiring re-ubiquitination by RNF126. The proteasome inhibitor bortezomib inhibits DENV and ZIKV replication. For ZIKV, this inhibition was linked to the substantial accumulation of ubiquitinated NS3 with impaired protease activity^95^. Accumulation of (misfolded) proteins can also trigger ER stress response and the PERK pathway that represses translation of components required for viral egress. Evidence supporting the potential of repurposing bortezomib to treat DENV infection was also provided in mice^96^. Other inhibitors of proteasomal degradation are shown in Table 1. For the role of ER stress-related UPR in DENV infection we refer to the review of Das and colleagues^39^.

**Table 1:** Post-entry host-directed DENV antivirals

Stage	Compound	DENV serotype	IC_50/ (90)_	SI:CC_50_/IC_50_	Reference
Translation	NITD451	DENV2	0.16 µM (A549)	>50	^44^
	Bromocriptine	DENV1 DENV2 DENV3 DENV4	1.6 µM (BHK 21) 1.2 µM 0.8 µM 0.8 µM	>33	^130^
	Lactimidomycin	DENV2	0.40 µM (IC_90_, Huh7)	/	^45^
	ST081006	DENV2	3.14 µM (Huh7)	8.03	^46^
	Narasin	DENV1 DENV2 DENV3 DENV4	0.65 µM (Huh7) 0.39 µM 0.44 µM 0.05 µM	>2500	^47^
	AR-12	DENV2	0.59 µM (A549)	142	^48^
	Catechin	DENV1 DENV2 DENV3 DENV4	37.94 µM (Huh7) 6.42 µM 38.95 µM 16.83 µM	>15.57 (DENV2)	^159^
Translocation	Cotransin 8 PS3061	DENV2	Active at 0.5 µM (Huh7 + C6/36), at 0.1 and 0.5 µM (MDDCs) Active at 1 µM (Huh7 + C6/36)	/	^53,66^
	Apratoxin S4	DENV2	0.003 µM (Huh7.5)	>300	^67^
Signal peptidase cleavage	Cavinafungin	DENV1 DENV2 DENV3 DENV4	0.004 µM (A549) 0.005 µM 0.003 µM 0.003 µM	160	^68^
Glycosylation/folding	NGI-1	DENV2	0.85 µM (HEK293T)	41	^76^
	Castanospermine	DENV2	85.7 µM (Huh7) 1 µM (BHK-21)	/	^77,79,81,160^
	Celgosivir	DENV1 DENV2 DENV3 DENV4	0.65 µM (BHK-21) 0.22 µM 0.68 µM 0.31 µM		
	Deoxynojirimycin derivatives, UV-4B	DENV1	5–38 µM (Vero)	>11.6–>477	^86,161^
		DENV2 DENV3 DENV4	6–22 µM 4–86 µM 3–18 µM		
	UV12	DENV2	22 µM (Vero)	>22	
	Iminocyclitol (36-37-38)	DENV2	4.7–11.8 µM (BHK-21) 9–18 µM (IC_90_)	>0.8–>2	^162^
	Sulfonium ions	DENV2	25.1–50.4 µM (Vero)	>3 >1	^163^
	Valiolamine derivates	DENV2	1.38 µM (Vero)	88	^90^
	AR-12	DENV2	0.59 µM	142	^48^
	JG40	DENV2	/(Huh7, C6/36)	/	^92^
ER rearrangement					
VP and CM biogenesis	CB-5083 NMS-873	DENV2luc.	0.25 µM (Huh7.5) 0.02 µM	100 1250	^117^
Cholesterol synthesis	Statins, Lovastatin	DENV2 DENV2luc.	>50 µM (K526) 1.8–46 µM (A549)	<1 <5	^104,107^ ^164^
	Atorvastatin	DENV2 DENV4	24.1 (Huh-7) 12.2 µM	2.35 1.67	
	Metformin	DENV2 DENV1 DENV2 DENV3 DENV4	3.82 µM (Huh7) 3108 µM (BHK-21) 4300 µM 3139 µM 3068 µM	8.61 /	^109,110^
	PF-429242 PF-06409577	DENV2	6.7 µM (Hela) 13.4 µM (Vero)	35 43.4	^111,115^
	Compound C	DENV2 HepG2	/	/	^112^
	Nordihydroguaiaretic Acid	/	/	/	^114^
	Hymeglusin	DENV2	4.5 µM (K526)	11	^104^
	U18666A	DENVluc.	6.2 µM (A549) 2.9 µM (Huh7)	11 12	^116^
Squalene synthesis	Zaragozic acid	DENV2	8.3 µM (K526)	6	^104^
Cholesterol transport	Ezetimibe	DENV1 DENV2 DENV3 DENV4	19.2 µM (Huh7) 13.4 µM 24.2 µM 17.7 µM	2.93 (DENV2)	^107,165^
Fatty acid synthesis	C75, Cerulenin	DENV2	~1 µM (Huh7)	/	^102^
	Orlistat	/	/	/	^166^
	PF-05175157, PF-05206574, PF-06256254	DENV2	1.0 µM (Vero) <1.3 µM 1.5 µM	236 319 135	^113^
RNA synthesis					
Inosine monophosphate dehydrogenase	Ribavirin,	DENV2	73 µM (Vero), 38 µg/ml, 40 µM (Huh7),	>13, / /	^141–143,167^
	ETAR, IM18, EICAR		9.5,106.1 µM (Vero), 1.6 µg/ml (Vero)	>105, / /	
	Mycophenolic acid	DENV2	0.3–1.9 (Vero, Huh7)	/	^143,167^
	N-allyl-acridone (3b)	DENV1 DENV2 DENV3 DENV4	26.7 µM (Vero) 13.5 µM 27.1 µM 12.5 µM	>37.3 >74.1 >36.9 >80	^144^
Dihydroorotate dehydrogenase	Brequinar	DENV2	0.078 µM (A549)	>320	^146^
	NITD-982	DENV2	0.005 µM (Vero)	>900	^147^
	Compound 3A	/	/	/	^148^
	AR-12, P12-23, P12-34	DENV2-eGFP	0.66, 0.07, 0.05 µM (A549)	33,297,388	^145^
	P12-34	DENV1 DENV2 DENV3 DENV4	0.10 µM (A549) 0.06 µM 0.09 µM 0.06 µM	206.3 326.5 223.4 317.8	
Assembly and Egress	C75	/	/	/	^153^
	SFV785	/	/	/	^157^
	Lovastatin	DENV2	38.4 µM (Vero)	1.4	^155^
	Nordihydroguaiaretic Acid	/	/	/	^114^
	Dasatinib	/	/	/	^150,156^
	Sunitinib	DENV1 DENV2 DENV3 DENV4	0.6 µM (BHK-21) 0.51 µM 0.3 µM 0.23 µM	>16.6 22.6 >33 >43	^158^
	Erlotinib	DENV1 DENV2 DENV3 DENV4	1.9 µM 2.5 µM 1.3 µM 3.9 µM	>10 >8 >15 >5	
	Castanospermine	DENV2	85.7 µM (Huh7) −1 µM (BHK-21)	/	^77^
	Hirsutine	DENV1	1.9 µM (A549)	>5.3	^168^
Ubiquitin proteasome pathway	Bortezomib	DENV2 DENV1-4	0.008 µM (BHK-21) <0.02 µM (primary monocytes)	/, >50	^95,96^
	MG132, ALLN	DENV2	0.4 – <10 µM (HepG2)	No tox at 0.4 and 10 µM	^169^
	CB-5083 NMS-873	DENV2luc.	0.25 µM (Huh7.5) 0.02 µM	100 1250	^117^
Ubiquitin-activating enzyme E1 (UBE1)	UBEI-41	/	/	/	^170^
Proteasome-associated deubiquitinating enzyme USP14	IU1	DENV2	40 µM (Hek293T)	7.5	^171^

DENVluc.: DENV-based replicon with structural genes replaced by Renilla, Firefly or nano luciferase.

### Flavivirus replication at ER-derived replication complexes

The newly translated NS proteins reside at the ER since flavivirus genome replication, as for other positive-stranded RNA viruses, is the next step to occur in close association with the ER membrane. The genome replication takes place in RCs, organelle-like structures in which viral NS proteins, viral RNA structures and cellular factors assemble (see Fig. 1C). These RCs provide an optimal environment for viral RNA replication by concentrating the factors that promote viral replication and by shielding viral RNA from cellular innate immune sensors. Electron tomography revealed that DENV infection induces ER membrane alternations of various morphologies, including invaginations into the rough ER lumen creating spherical vesicles (of 90 nm diameter) and bundling of smooth ER membranes (CM, convoluted membranes). Detection of double-stranded RNA and NS proteins inside invaginated vesicles indicated that these are the sites of active viral RNA replication. The vesicles stay connected with the cytosol by means of small pores that allow exchange of nucleotides and RNA molecules^24,97^.

The transmembrane proteins NS4A and NS4B are considered as the main drivers in the formation of the RC^98^. A model was proposed in which the TMDs and intramembrane domains of both NS4A and NS4B function as a wedge to induce invaginated vesicles with negative membrane curvatures that are positioned at the lumenal side of the ER. This negative membrane curvature is stabilized by oligomerisation and interaction of NS4A and NS4B^24^. NS1 dimers interact with NS4A and NS4B at the luminal side and hydrophobic residues of NS1 could insert into the lumenal leaflet of the ER membrane to introduce membrane bending, assisting in the formation of invaginated vesicles^25,98^. NS2A, another transmembrane protein with five TMD α helices, was also suggested to contribute to these membrane rearrangements and to stabilize the pore-like opening^24^. Besides the viral NS proteins, several host factors are involved in this ER membrane remodelling. Silencing of the cellular host factors reticulon and atlastin impaired membrane invagination and subsequent viral replication. Both proteins are hairpin-like transmembrane proteins that can form a wedge-like structure embedded in the ER membrane at the cytoplasmic leaflet to induce membrane deformations^98^. Reticulon is recruited to the RC to assist in remodelling of the host membranes and to stabilize the viral proteins NS4A and NS4B in the ER. However, it should be noted that the reduced stability of NS4B in the absence of reticulon was solely demonstrated through ectopically expressed WNV NS4B-GFP, but not in DENV- and WNV-infected cells^99^. DENV is also able to hijack more divergent host proteins, like cytoskeletal component vimentin. The N-terminal cytoplasmic region of DENV NS4A interacts with host vimentin to anchor RCs in the ER^100^. As previously stated, a direct interaction was also reported between OST with flaviviral NS1 and NS4B, and with NS2A, NS3 and NS4A, pointing to a critical structural role for the OST in the generation of a functional RC^49,72^.

To acquire the replication platform and to generate a correct membrane curvature, flaviviruses have the capacity to expand the ER membrane and modify its composition to increase fluidity. The membranes at the sites of replication are probably enriched in cholesterol, phospholipid, sphingolipid and fatty acid. Albeit the ER constitutes the site for synthesis of these components, the ER membranes have low cholesterol and sphingolipid content themselves due to transport of these lipids to other subcellular organelles. Hence, flavivirus replication requires adaptation of the lipid biosynthesis. Fatty acid synthesis is upregulated in flavivirus-infected cells and DENV NS3 recruits fatty acid synthase complex to the sites of replication. DENV also depends on acetyl-Coenzyme A carboxylase, another key enzyme in the fatty acid synthesis^101–103^. In addition, the synthesis of more complex lipids like cholesterol is essential for flavivirus replication. 3-Hydroxy-3-methylglutaryl-CoA reductase (HMGCR), a rate-limiting enzyme catalysing the second step in the cholesterol biosynthesis by the mevalonate pathway, is recruited to the replication sites and its activity is enhanced through inactivation of 5′AMP-activated protein kinase (AMPK) during DENV infection^24,103^. Besides the de novo cholesterol biosynthesis, DENV also promotes the uptake of low-density lipoprotein by upregulated expression of its receptor and the cholesterol transporter Niemann-Pick C1-Like 1 (NPC1L1) receptor on the surface of infected liver cells^103^.

The dependency on the lipid content of the ER for flavivirus replication (but also for entry, fusion, and assembly) is further evidenced by abolished infection of DENV upon pharmacological inhibition of lipid biosynthesis. Statins are the main drugs to interfere with the cholesterol biosynthesis pathway through inhibition of HMGCR^103^. In vitro studies have revealed that statins can counteract flavivirus infection, in cancer cell lines (A549) with inhibition of cell growth but no cytotoxicity was observed in primary peripheral blood mononuclear cells (PBMCs). Statins were shown to affect the formation of RCs^104,105^. In addition, statins had a protective effect against DENV infection in AG129 mice^106,107^. Despites this evidence, the repurposed lovastatin showed no beneficial effect on viremia and clinical manifestations related to DENV infection in adult patients^108^. DENV is also susceptible to ezetimibe which interferes with cholesterol transport by blocking NPC1L1. Ezetimibe reduced the percentage of Huh7-infected cells and significantly increased the survival of AG129 mice. Regardless of the synergetic effect (against DENV2) or the additive effect (against DENV4) of a combinational treatment of ezetimibe and atorvastatin in vitro, their anti-DENV effect was lost if this combination was administered in vivo^107^. On the other hand, activation of AMPK can inhibit DENV replication as explained above. The use of metformin as an DENV antiviral, a diabetes drug that indirectly activates AMPK, has led to contradictory results depending on the cell type, animal model and DENV strain^109,110^. Nevertheless, preliminary results suggest that metformin lowers the risk of severe DENV in diabetic patients^110^. In addition, treatment with a direct activator of AMPK, PF-06409577 inhibited DENV2 multiplication with a better selectivity index (SI) related to less off target effects^111^, while the inhibition of AMPK by Compound C exacerbated DENV infection^105^. However, an independent study reported an inhibitory effect on DENV infection for Compound C. These contradictory results imply that DENV replication relies on a tightly adjusted regulation of AMPK function^112^. For a complete overview regarding the role of AMPK in DENV infection we refer to the review of Farfan-Morales et al.^103^. Also, inhibitors of fatty-acid synthase and acetyl-Coenzyme A carboxylase can be repurposed as broad-flaviviral drugs. Inhibitors of acetyl-Coenzyme A carboxylase (like PF-05175157, PF-05206574, and PF-06256254) showed promising levels of potency against DENV (see Table 1)^113^. However, the main side effects of fatty acid synthase inhibitors are severe anorexia and weight loss^42^. Finally, flaviviruses are susceptible to nordihydroguaiaretic acid which interferes with transcription factor sterol responsive element binding protein (SREBP) of cholesterol and fatty acid synthesis, thus affecting both pathways^114^. Immature SREBPs are activated by site-1 protease enzyme upon cleavage. The small competitive inhibitor of site-1 protease PF-429242 (dihydrochloride) exerted pan-serotype activity. However, viral propagation was not restored by addition of exogenous fatty acids or cholesterol, suggesting a different yet unknown anti-DENV mechanism^115^. Furthermore, an additive antiviral effect was observed for the co-treatment of the cholesterol transport inhibitor U18666A and the fatty acid synthase inhibitor C75^116^. It should be noted that drugs targeting lipid biosynthesis can also affect other steps of the flavivirus life cycle besides RC formation (such as entry, fusion, assembly and egress) and induce several non-cholesterol related effects, such as anti-inflammatory and immunomodulatory properties as reviewed by Farfan-Morales and colleagues^103^. It needs to be taken into consideration that manipulation of a major metabolic pathway such as lipid biosynthesis could induce severe side effects in the host.

### Convoluted membranes (CMs)

CMs, the other membrane rearrangements induced in DENV-infected mammalian cells (but not in infected mosquito cells) are interconnected with RCs within the same endomembrane network^97,101^. CMs were believed to be the sites of polyprotein synthesis, processing, and storage. However, since no ribosomes are detected here, CM are now believed to be the sites of lipid storage^97,98^. Within the CM, NS4B associates with VCP. VCP is a ubiquitous host protein and contributes to ER- and mitochondria-associated degradation by retro-translocation of misfolded membrane proteins to the cytosol and targeting of these substrates to the proteasome. Its role in protein homeostasis is discussed above. In the context of CMs, VCP may be physically recruited to CMs and directly regulate vesicle morphogenesis through its interaction with NS4B. Furthermore, the de novo vesicle biogenesis depends on the ATPase activity of VCP. Consistently, the VCP inhibitor CB-5083 reduces the formation of CMs and spherical vesicles/RCs in DENV-infected cells. Thus, VCP provides an attractive broad-spectrum antiviral target in drug repurposing approaches given that new VCP-targeting drugs are currently in clinical trials for cancer treatment^117^.

### Flaviviral RNA synthesis at the replication complexes

All viral NS proteins are components of the RC. Transmembrane proteins NS2A, NS2B, NS4A and NS4B impregnate the lipid bilayer and dimers of NS1 protein stabilize the RC from the luminal side of the ER through interactions with NS4A and NS4B providing a shielded compartment for the viral RNA, NS3 helicase and NS5 polymerase (see Fig. 1C)^24^. NS3 and NS5 carry out all enzymatic activities to fulfil RNA synthesis, RNA capping and methylation. Complementary sequences in the 5′ and 3′ UTR can cyclize through base pairing, an essential event to initiate synthesis of the negative strand. Starting from the genomic positive RNA strand as template, NS5 synthesizes an uncapped negative RNA strand generating a dsRNA intermediate. The dsRNA intermediate is unwound by NS3 helicase assisted by NS4B^118,119^. The negative strand in the dsRNA intermediate serves as a template for RNA amplification to produce multiple copies of positive RNA strands (asymmetric replication) which are capped at the 5′ end. The RNA capping process depends on three enzymatic activities. First, the 5′ triphosphatase activity of NS3 dephosphorylates the 5′ triphosphate end of (+) genomic RNA strands. Next, a guanine cap is created by transfer of a guanosine monophosphate to diphosphorylated RNA by NS5 guanylyltransferase. The guanine cap is finally methylated in two steps to generate a mature m7GpppAm type 1 cap by methyltransferase activities of NS5 using *S-*adenosyl-L-methionine (AdoMet) as a methyl donor^118^. The newly synthesized and capped RNA can be incorporated into newly formed particles, which also assemble on the ER membrane via budding and encapsidation within the cisternae opposite to the pores of the vesicles. Virions travel to distal sites in the ER lumen where they accumulate in dilated ER cisternae^97^.

So far, most of the research on flavivirus-targeting antivirals has focussed on the enzymatic activities of NS3 and NS5 at the RC, as well as on the E protein (see Table 2). Numerous nucleoside analogues have been identified in anti-flaviviral screenings, exhibiting activity in vitro and in vivo against multiple flaviviruses. However, most of these nucleoside analogues failed in (pre-)clinical stages because of low efficacy or high toxicity (balapiravir, NITD008)^120,121^. The only nucleoside analogues still under investigation in clinical trials are the guanosine analogue, AT-752 and its prodrug AT-281 which are currently in a phase 1 (NCT05366439) and phase 2 clinical trial (NCT05466240) to assess safety and anti-DENV activity^11^. An explanation for the limited success of nucleoside inhibitors is their dependency on host kinases for their phosphorylation and their inhibitory effect on off-target cellular polymerases^42^. On the other hand, a few non-nucleoside inhibitors of NS5 have been identified that allosterically hinder NS5 by targeting the N-pocket or the RNA tunnel (see Table 2)^122–124^.

**Table 2:** Post-entry virus-directed DENV antivirals

Stage	Compound	DENV serotype	IC_50/ (90)_	SI:CC_50_/IC_50_	Reference
5′ and 3′ stem loop	Peptide-conjugated phosphorodiamidate morpholino oligomers	DENV2	1 µM (BHK)	25	^41^
NS2B-NS3 protease cleavage	Benzyl ethers of 4-hydroxyphenylglycine (compound 104)	DENV2	3.4 µM (Huh-7)	>25	^172^
	Boronic acids analogues (compound 7)	Anti-NS2B-NS3	18 µM	>5	^173^
	Thiazolidinone-peptide hybrid 10a	DENV2luc.	16.7 µM (Huh-7)	>3	^174^
	N-sulfonyl-peptide-hybrids	Anti-NS2B-NS3	0.078 µM	/	^175^
	Phenylalanine analogues (Compound 45a)	Anti-NS2B-NS3	0.26 µM	/	^176^
	Diaryl(thio)ethers (Compound 1–6, 8)	DENV2	0.1–3.5 µM (Vero)	±10	^177^
	Protegrin-1	Anti-NS2B-NS3	11.7 µM	/	^178^
	Retrocyclin-1	Anti-NS2B-NS3	21.4 µM	/	^179^
	Flavonoids	Anti-NS2B-NS3	21 µM	/	^180^
	ARDP0006	DENV2	4.2 µM (LLC-MK2)	16.6	^181^
	BP13944 BP2109	DENV2luc.	1.03 µM (BHK-21) 0.17 µM	70 172	^182,183^
	Tolcapone (Compound F)	DENV2	2.03 µM (BHK-21)	14.36	^184^
	166347	DENV1 (anti-NS2B-NS3) DENV2 DENV3 DENV4	3 µM 5 µM 5 µM 11 µM	/	^185^
	SK-12	DENV1 DENV2 DENV3 DENV4	0.97 µM (Vero) 0.98 µM 2.43 µM 0.74 µM	69 69 28 91	^186^
	Policresulen	DENV2luc.	4.99 µg/ml (BHK-21)	92	^187^
	Palmatine	DENV2	26.4 µM (Vero)	39	^188^
	Nelfinavir	DENV2	3.5 µM (Vero-B)	4.6	^70^
	ABT263, ABT737, AT101, and TW37	Anti-NS2B-NS3	0.86, 1.15, 0.81, and 0.89 µM	/	^71^
NS3 helicase activity	Suramin	Molecular beacon Helicase assay	0.4 µM	/	^125^
	ST-610	DENV1 DENV2 DENV3 DENV4	0.24 µM (Vero) 0.27 µM 0.26 µM 0.20 µM	>370	^128^
	Ivermectin	DENV2	0.7 µM (Vero)	5.42	^129^
	Compound 24 (benzothiazole) Compound 25 (pyrrolone)	DENV2luc.	7.1 µM (BHK-21) 36 µM	17 4.5	^189^
NS5 methyltransferase activity	AT-752, AT-281	DENV1luc. DENV2luc. DENV3luc. DENV4luc.	0.57 µM (Huh7) 0.30 µM 0.75 µM 0.57 µM	>298 >566 >226 >298	^131^
	Azidothymidine based thiazoles (compound 11i)	DENV2luc.	7.5 µM (BHK)	2.9	^190^
	NSC 12155	DENV2	7.0 µM (A549)	13.7	^191^
	Sinefungin	DENV MTase activity	0.03–0.04 µM	/	^192^
NS5 guanylyltransferase	BG-323	DENV2luc.	30.8 µM (BHK)	6.0	^193^
NS5 polymerase activity	Balapiravir (R1479)	DENV1-4	1.9–11 µM (Huh7)	/	^120^
	NITD008	DENV2	0.64 µM (Vero) 1.64 µM (Huh7)	>156 >61	^121^
	AT-752, AT-281	DENV1luc. DENV2luc. DENV3luc. DENV4luc.	0.57 µM (Huh7) 0.30 µM 0.75 µM 0.57 µM	>298 >566 >226 >298	^131^
	7-deaza-2-C-methyl-adenosine	DENV2	15 µM (Vero)	>21	^194^
	Compound 29 (acyl-sulfonamide derivatives)	DENV2luc.	1.9 µM (Huh7)	>26	^122^
	Compound 27 (biphenyl acetic acid)	DENV1 DENV2 DENV3 DENV4	1.8 µM (A549) 2.3 µM 1.8 µM 1.8 µM	>27 >21 >27 >27	^123^
	NITD-434 NITD-640	DENV2	31.2 µM 10.7 µM	>1.6 >4.7	^124^
NS3-NS4B complex formation	JNJ-A07 JNJ-1802	DENV1-4 DENV2	≤0.006 µM (Vero) 0.000059 µM (Vero)	130.000 44.000	^132,133^
NS4B	NITD-681	DENV1 DENV2 DENV3 DENV4	1.5 µM (BHK-21) 1.6 µM 1.6 µM 4.1 µM	>9.8	^135^
	NITD-688	DENV1-4 DENV2	0.008–0.038 µM (A549) 0.007 µM (Huh7)	≤732 4171	^136^
	Lycorine	DENV1-2	/	/	^137^
	Spiropyrazolopyridone and derivatives (1a)	DENV2	0.012 µM (HepG2)	>833	^138^
	SDM25N	DENV2luc.	1.9 µM (Hela)	>5	^139^
	AM404	DENV2luc.	3.6 µM (Hela)	>7	^140^

DENVluc.: DENV-based replicon with structural genes replaced by Renilla, Firefly or nano luciferase.

The NS3 helicase activity is driven by an intrinsic nucleoside triphosphate activity. Several inhibitors of the NS3 helicase have been reported. A molecular beacon helicase assay identified suramin as a potent non-competitive inhibitor of DENV NS3 helicase^125^. However, suramin has been reported to inhibit numerous other enzymes (such as helicases, polymerases, reverse transcriptase, telomerases, metabolic enzymes and several others) and can therefore inhibit different viruses (e.g., ZIKV, Chikungunya virus, Hepatitis virus, Ebola virus, HIV, Herpes simplex virus, and Enterovirus) indirectly at several stages of the infection cycle. Also, various adverse effects can be linked to the non-specific nature of suramin^126,127^. ST-610, a benzoxazole analogue, exerts pan-serotype activity inhibiting the helicase activity without inhibiting the nucleoside triphosphatase activity. ST-610 significantly reduced viremia in the sublethal DENV murine model^128^. In addition to its weak anti-NS3 protease activity and other reported effects, ivermectin also inhibits NS3 helicase in the high nanomolar range (0.5 µM)^129^. The small molecule bromocriptine inhibits DENV1-4 replication although NS3 escape mutants have been observed. However, NS3 enzymatic activities were not affected in the presence of bromocriptine and escape mutants conferred partial drug resistance suggesting the involvement of other viral or host factors^130^.

As expected, blocking the methyltransferase and guanylyltransferase activity of DENV NS5 (involved in the 5′ capping of viral RNA) has also been shown to block DENV propagation (see Table 2). For example, the nucleoside analogue AT-281 targets, besides the elongation during RNA synthesis, also the 2′-O methylation step during viral capping^131^. The primary drawback associated with inhibitors targeting the enzymatic activities of NS3 and NS5 is their relatively limited selectivity for the viral enzymes in respect to the host enzymes with similar functions^42^.

A recent breakthrough in the antiviral research against flaviviruses is the identification of the highly potent pan-flavivirus inhibitor JNJ-1802 that blocks the NS3-NS4B interaction within the viral RC. JNJ-1802 showed activity in vivo in mice and in non-human primates and was found safe and well-tolerated in phase 1 clinical trials. Furthermore, the drug was found to have a high barrier to resistance^132^. Resistance studies with an analogue JNJ-A07 suggest that the inhibitors allosterically alter the confirmation of the cytosolic loop 3 of NS4B^133^. As described earlier, the NS3-NS4B complex has an important role in the RC, where NS4B dissociates NS3 from the viral RNA and supports its helicase activity^134^. Several other NS4B inhibitors were identified using phenotypic screenings^135–140^.

Flaviviruses rely on the de novo cellular synthesis of nucleotides in the host cell for their replication. Host inosine monophosphate dehydrogenase (IMPDH), the first enzyme in the cellular synthesis of guanine nucleotides, has been shown to act as a drug target for flavivirus inhibition (see Table 1). The guanosine analogue ribavirin was one of the first approved broad-spectrum antivirals by IMPDH inhibition. However high concentrations of ribavirin are required to exert anti-flavivirus activity in vitro. Meanwhile, derivates of ribavirin (ETAR, EICAR) have been developed with more potent anti-DENV activity^141,142^. Likewise, the more potent non-nucleoside inhibitor mycophenolic acid has broad anti-flavivirus activity but is limited by immune-related cytotoxicity^42,142,143^. A pan-serotype inhibitor with higher specificity to block viral replication in comparison to cellular viability (no toxicity at 1 mM) is the N-allyl acridone derivate 3b. IMPDH appears to be partially involved in the anti-DENV activity of derivate 3b, in addition to a yet unidentified mode-of-inhibition. This dual mechanism of action may explain the lower cellular toxicity^144^. Furthermore, brequinar and AR-12 derivatives were shown to inhibit dihydroorotate dehydrogenase (DHODH), an enzyme of cellular pyrimidine synthesis in the mitochondria. Potent broad anti-flavivirus activity was assigned to these compounds in vitro and AR-12 derivatives improved survival in mice subcutaneously challenged with DENV. In case of brequinar, a low therapeutic index halted it from proceeding to clinical trials^42,145,146^. NITD-982, another potent inhibitor of DHODH is active against DENV in vitro but lacked efficacy in DENV-AG 129 mice^147^. Compound 3A, which is supposed to deplete the pyrimidine pools, is an interesting small molecule because of its broad-spectrum antiviral activity (positive and negative strand RNA viruses, DNA viruses and retroviruses) in combination with low cytotoxicity^148^. Compound 2 and 3 in the study of Ye et al. inhibited S-adenosylhomocysteine hydrolase, an enzyme involved in the synthesis of adenosine^149^. Other potential targets of host directed antivirals are cellular kinases used by viruses throughout their life cycle, as DENV NS5 was shown to be phosphorylated by host kinases. The membrane-associated Fyn tyrosine kinase has a critical role in DENV RNA replication as suggested by the reported anti-DENV effect of the Fyn kinase inhibitors AZD0530 and dasatinib^150^.

### Assembly and virion budding

In general, viral protein synthesis, RNA replication and virion assembly are profoundly connected. For flaviviruses the viral particles are formed by budding of nucleocapsid structures, composed of Ca proteins that are bound to genomic RNA strains, and surrounded by ER-derived membranes that carry the prM and E proteins^98^. Electron microscopy studies revealed that virion assembly occurs predominantly in regions of the ER opposite to the viral RCs^97^. The unique physical architecture of the ER with its layered sheets makes such an intimate spatial arrangement possible and facilitates RNA incorporation into virion particles while protecting the viral RNA from the host immune response by isolating the RNA replication and virion assembly interface from the cytosol^151^. The viral RNA, NS2B-NS3 protease and the structural proteins are recruited by NS2A to the site of assembly. The 3′ UTR of the viral RNA contains a “recruiting signal for packaging” that binds to a cytosolic loop of NS2A allowing NS2A to recruit nascent RNA from the RC to the virion assembly site^19^. The Ca-prM protein is sequentially processed by the viral protease to release the Ca protein which induces a change at the ER lumenal SPase cleavage site, followed by efficient SPase cleavage. The outcome of sequential processing is the delay of prM production required for efficient nucleocapsid incorporation^152^. The Ca protein localizes on ER-derived lipid droplets indicating that lipid droplets are involved in viral genome packing. Pharmacological manipulation of lipid droplet formation in the cell with the fatty acid synthase inhibitor C75, had a profound suppressive effect on viral particle production^153^. Small vesicles containing the assembled proteins are pinched off in the lumen of the ER and accumulate before travelling to the Golgi apparatus^154^.

The DENV envelope originates from modified portions of the ER consisting of glycerophospholipids, sphingolipids, fatty acids and cholesterol to confer stability and robustness to the virions. The amount of sterol within the virions is critical for their infectivity^103^. For that reason (lova)statins may also exert anti-DENV activity through interfering with the assembly step^155^. Likewise, dasatinib was shown to affect assembly in addition to replication. Like its replication inhibition, which is supposed to depend on Fyn kinase, its inhibitory effect on the assembly may also depend on the Src-family of kinases^150,156^. Other kinases were shown to be involved in the recruitment and assembly of the nucleocapsid in ER-specific compartments. Administration of the protein kinase inhibitor SFV785 results in secretion of viral particles devoid of dense nucleocapsids, pointing to a role for NTRK1 and MAPKAPK5 kinases in the assembly of flaviviruses^157^. Moreover, the two host cell kinases AP2-associated protein kinase 1 (AAK1) and cyclin G-associated kinase (GAK), which regulate the trans-Golgi network (TGN) transport, contribute to flavivirus assembly/release, as evidenced by the reported antiviral effects of their inhibitors, the approved anti-cancer drugs sunitinib and erlotinib. The expression of AAK1 correlates with intracellular virus abundance, suggesting that targeting overexpressed kinases, as commonly done in cancer treatment, may facilitate suppressing viral replication. Furthermore, in vivo studies in mice suggest that it might be feasible to use a safe therapeutic dose for sunitinib and erlotinib to treat flaviviral infections^158^.

To facilitate their budding from the endosome, flaviviruses appear to usurp the endosomal sorting complex required for transport (ESCRT), as its depletion reduced the number of infectious viral particles^154^. Flaviviruses are delivered to the Golgi apparatus as immature virions. The pr segment of the prM protein covers the fusion loop of the E protein to prevent premature viral fusion of endosomal membranes during egress. Exposure to the acidic environment of the Golgi and TGN triggers conformational changes in the prM and E protein making the prM accessible for cleavage by TGN protease furin. As a result of this proteolytic event, mature M protein is separated from its pr segment which remains non-covalently attached to further protect against premature fusion. The pr segment only dissociates from the viral envelop upon the acidic to neutral pH shift in the extracellular space, resulting in the ultimate release of infectious mature virions^14^.

## Conclusion

Undoubtedly, the ER is the key organelle of the host during DENV infections, supporting various stages throughout the entire life cycle of flaviviruses. It serves as a pivotal hub in the flavivirus infection cycle, accommodating viral protein translation and maturation, membrane insertion, viral replication, virion assembly and initiation of the transport of newly formed viral particles via the secretory pathway for their egress. By subcellular localization within ER-derived membrane structures during different stages of the replication cycle, DENV is able to overcome competition with host cell proteins to warrant efficient viral replication and to escape from cellular innate immune sensors. Consequently, the ER presents a multitude of potential sites for anti-flaviviral drug development. Several promising host-directed ER-related drugs and direct virus-targeting antivirals have been reported in the last decade, with a few of them entering clinical trials. Attention should be given to re-evaluating the flavivirus replication inhibitory potency of the arsenal of clinically approved drugs, e.g., those that interfere with ER-related events such as UPR or ER stress. Given the escape potential of RNA viruses, and flaviviruses in particular, it is also advisable to invest in antiviral strategies that combine a viral target with a host cell factor. Future molecular research exploring the intricate interplay between the host cell ER and DENV may probably unveil new therapeutic target sites, offering opportunities to develop additional selective anti-DENV drugs.
